# Aquatic ecosystem health assessment of a typical sub-basin of the Liao River based on entropy weights and a fuzzy comprehensive evaluation method

**DOI:** 10.1038/s41598-019-50499-0

**Published:** 2019-10-01

**Authors:** Jiabo Chen, Yanjie Wang, Fayun Li, Zicheng Liu

**Affiliations:** 1 National & Local United Engineering Laboratory of Petroleum Chemical Process Operation Optimization and Energy Conservation Technology, Liaoning Shihua University, Fushun, 113001 China; 2 Institute of Eco-environmental Sciences, Liaoning Shihua University, Fushun, 113001 China; 3 Dalian Environmental Monitoring Centre, Dalian, 116023 China

**Keywords:** Ecology, Environmental sciences

## Abstract

River ecosystem health assessments provide the foundation for river ecological protection and integrated management. To evaluate the aquatic ecosystem health of the Fan River basin, benthic macroinvertebrate indices (the Multimeric Macroinvertebrates Index Flanders (MMIF) and Family Biotic Index (FBI)), a habitat index (the river habitat quality Index (RHQI)) and a water quality index (the Improved Water Pollution Index (IWPI)) were selected. The entropy weighting method was used to calculate the RHQI and IWPI. A fuzzy comprehensive evaluation method was used to evaluate the aquatic ecosystem health. The evaluation results indicated that the aquatic ecosystem health of the Fan River basin was better in 2018 than in 2011, which respectively belonged to the ends of the 11th and 12th Five-Year Plans of the Major Science and Technology Programs for Water Pollution Control and Treatment in China. The proportions of sampling stations with good, moderate and poor grades in 2011 were 50.0%, 40.0% and 10.0%, respectively, and in 2018, the proportions of stations with excellent, good and moderate grades were 20.0%, 50.0% and 30.0%, respectively. A correlation analysis showed that the RHQI was significantly correlated with the MMIF, FBI and IWPI. The riparian land use pattern was an important factor that influenced changes in the aquatic ecosystem health grade. Of the water quality parameters, total phosphorous (TP) and potassium bichromate index (COD) were the main factors that affected the characteristics of benthic macroinvertebrates and the aquatic ecosystem health.

## Introduction

In recent years, the utilization of water resources has increased, and aquatic ecosystems have been seriously degraded or destroyed. With the goal of achieving a healthy ecological environment, increasing attention has been paid to the health of aquatic ecosystems^1^ based on integrated biological, water quality and habitat indicators^2–5^. The health of aquatic ecosystems comprehensively reflects the physical, chemical and biological integrity and spatial differentiation characteristics of river ecosystems and can effectively reflect the structure and function of the ecosystems^6^.

In this respect, because the characteristics of benthic macroinvertebrates can reflect the extent of damage to aquatic ecosystems at different scales in a basin, macroinvertebrates are now among the most important biological indicators of watershed and aquatic ecosystem health^6^. Macroinvertebrates have been used as bioindicators for integrative measures of ecosystem health in rivers due to their advantages of limited mobility, large-scale applicability, long lifespan and broad distribution compared with other physicochemical hydrological parameters^7–9^. Previous studies have shown that macroinvertebrate species vary in their sensitivity to different types of pollutants and can reflect the occurrence of intermittent or unrecorded chemical pollution incidents^10,11^. Moreover, macroinvertebrate species traits are sensitive to disturbances and can be used to identify functional relationships with important environmental factors^12,13^. Ephemeroptera, Plecoptera and Trichoptera among benthic macroinvertebrates are greatly influenced by dissolved oxygen and sediment types. A stone substrate with different particle sizes is beneficial to the survival of climbing macroinvertebrates. The dissolved oxygen is an important condition for the survival of macroinvertebrates. When the dissolved oxygen concentration is high, the proportion of aerobic macroinvertebrate group is large. When there are no pebbles or gravel of different sizes in the substrate and the dissolved oxygen concentration is low, the proportion of the Ephemeroptera, Plecoptera and Trichoptera (EPT) groups decreases or even disappears. Gastropods, oligoptera and some diptera are known benthic macroinvertebrates that can tolerate unfavorable conditions and survive with low dissolved oxygen and high pollutant concentrations (COD and NH_4_^+^-N). Assessments of disturbances to rivers are often based on the collection and analysis of benthic macroinvertebrates to provide a measure of the health of the river ecosystem^14^.

River ecosystem health assessments include the habitat characteristics of the river sediment, hydrological conditions^15^, water quantity and flow velocity, vegetation structure and coverage, riparian type^2^, erosion degree, land use patterns^16^, and other factors. Human activities, such as water conservation and engineering projects, mining, reclamation, riverside agriculture, urbanization and others, can significantly impact the river environment^17^. These human activities often destroy the continuity of rivers, cut off the connection between upstream and downstream reaches, and change the types and characteristics of rivers. Notably, the flow velocity, nutrient load, sediment deposition rate, and water level may all change, thereby directly or indirectly affecting the survival of aquatic organisms, water quality, and the health of the river ecosystem^18^.

Water quality is an important component of a water ecosystem health assessment. The increased deterioration of water quality can be a result of various human disturbances and anthropogenic pressures, such as rapid population growth, industrialization, the expansion of urban and suburban areas, wastewater discharge, non-point source pollution (e.g., domestic, sewage and agricultural sources), land use change, and the removal of riparian vegetation^10,11,19–22^. The pollutants enter river ecosystems through land drainage, surface runoff and precipitation and can result in serious ecological problems. Pollution problems in rivers can greatly reduce habitat heterogeneity and water quality and directly and indirectly affect aquatic organisms in the catchment^23^, leading to adverse impacts on the river ecosystem^24^.

The most common methods of river health assessment mainly include predictive modeling methods and multi-index evaluation methods^25–28^. In multivariate assessment methods^29,30^, river health assessments generally use the corresponding environmental parameters and biological indicators^31^ as the basis for evaluation through index calculations. Based on the benthic macroinvertebrate characteristics, habitat and water quality, suitable indices and parameters are selected to evaluate the quality of aquatic ecosystems and obtain assessment results based on comprehensive calculations and analyses, such as by fuzzy comprehensive evaluation methods. By combining fuzzy comprehensive evaluation methods with the river ecological health evaluations, comprehensive quantitative calculations and analyses can be performed based on the indices of hydrological and ecological characteristics. In this approach, the calculation process and results are objective. The evaluation results can directly reflect the ecological health status of a river, and multiple indices should be considered to avoid the bias of a single index evaluation. Of the biological indices of benthic macroinvertebrates, the Multimeric Macroinvertebrates Index Flanders (MMIF) and Family Biotic Index (FBI) were selected in this study; these indices can adequately express the community composition, diversity, sensitivity, tolerance and individual distribution of macroinvertebrates^32,33^. The river habitat quality Index (RHQI) and Improved Water Pollution Index (IWPI) based on the entropy weighting method were also used to classify the river health^34,35^. The goals of the study were to investigate and classify aquatic ecosystem health based on the above four indices and to investigate the potential links among benthic macroinvertebrates, habitat characteristics and water quality. Aquatic ecosystem health assessments have been increasingly used for conservation improvements and the integrated management of hydroecology and restoration actions because they provide relevant information and an important scientific basis for regulatory agencies and decision makers. In the study area, health assessments based on multimeric methods are rare, highlighting the need for an effective hydroecological management strategy in the area.

## Results and Discussion

### Aquatic ecological characteristics of the Fan River basin

#### Characteristics of benthic macroinvertebrates

As ubiquitous and effective indicators, benthic macroinvertebrates are often the organisms of choice for biomonitoring to identify anthropogenic pressures, such as river habitat alterations and water pollution^36,37^. In total, 57 benthic macroinvertebrate taxa were identified from macroinvertebrate samples collected from the 10 sites in the Fan River basin in 2011 and 2018. All collected macroinvertebrate samples belonged to three phyla (Annelida, Mollusca, and Arthropoda) and 11 orders (Diptera, Ephemeroptera, Trichoptera, Plecoptera, Coleoptera, Hemiptera, Decapoda, Basommatophora, Gnathobdellida, Rhynchobdellida, and Plesiopora). In general, macroinvertebrate communities were mainly dominated by aquatic insects, especially Ephemeroptera, Trichoptera and Diptera.

**Figure 1:**
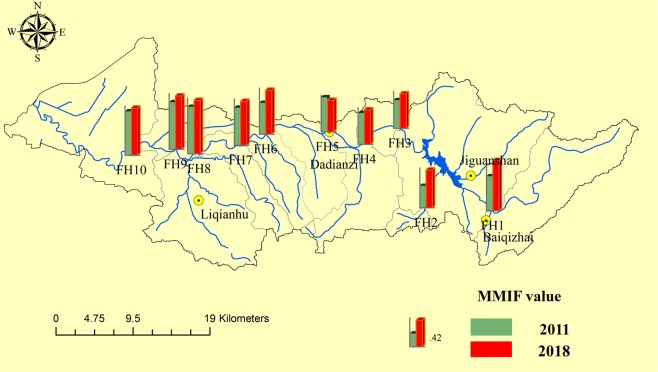
MMIF value of the Fan River basin in 2011 and 2018.

**Figure 2:**
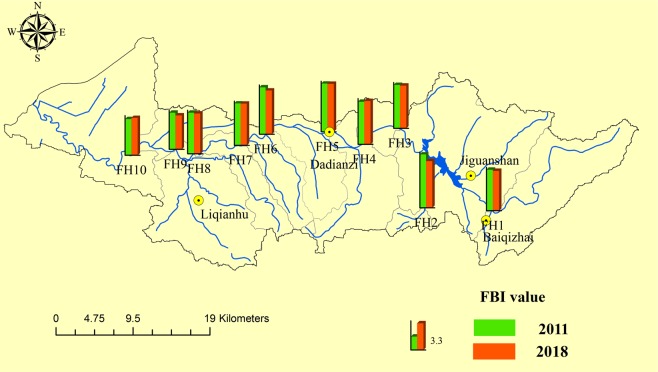
FBI value of the Fan River basin in 2011 and 2018.

Two biological metrics, MMIF and FBI, which are based on macroinvertebrate measures and are commonly used in river ecological health assessments, were calculated for all the sampling stations (Figs 1 and 2). The MMIF integrates different biological measures (such as the taxa richness, EPT taxa abundance, sensitive taxa abundance, Shannon-Wiener diversity and mean tolerance score) into a single value^32^ that adequately reflects the characteristics of the benthic macroinvertebrate community structure and the effects of multiple anthropogenic pressures.

The tolerance scores of the families/genera of macroinvertebrate taxa in the study area were listed in Table 1. The tolerance scores are between 1 and 10, where 10 means very sensitive to pollution and 1 means strong tolerance to pollution. The 57 taxa belonged to 36 families/genera, and 32 of the families/genera were the same as those in ref.^32^. According to the comparison of the list of families and genera of macroinvertebrate taxa, 4 taxa were not included in ref.^32^, namely, *Cincticostella* and *Drunella* of Ephemeroptera, Stenopsychidae of Trichoptera, and *Galba* of Mollusca. The characteristics, sensitivity, tolerance scores and living environment requirements of these four taxa were verified by refs^38,39^. The tolerance scores of the 4 different families/genera were also fully verified according to the relevant references and met the requirements of the MMIF index.

**Table 1:** The tolerance scores of the families/genera of macroinvertebrate taxa in the Fan River basin, ranging from 10 for very sensitive to pollution to 1 for very tolerant to pollution.

Taxon	Tolerance Scores
**Ephemeroptera**	
*Baetis*	6
*Caenis*	6
*Cincticostella*	10
*Drunella*	10
*Epeorus*	10
*Ecdyonurus*	9
*Ephemera*	8
*Paraleptophlebia*	8
*Siphlonurus*	7
**Trichoptera**	
Hydropsychidae	6
Limnephilidae	8
Rhyacophilidae	8
Stenopsychidae	8
**Plecoptera**	
*Isoperla*	10
*Perlodes*	10
**Diptera**	
Ceratopogonidae	3
Chironomidae	
-non thummi-plumosus	3
-thummi-plumosus	2
Ephydridae	3
Simuliidae	5
Tabanidae	3
Tipulidae	3
**Coleoptera**	
Dytiscidae	5
Haliplidae	6
**Odonata**	
*Gomphus*	7
**Hemiptera**	
Callicorixa	5
**Crustacea**	
Gammaridae	5
Palaemonidae	5
**Mollusca**	
*Galba*	5
*Gyraulus*	6
*Lymnaea*	5
*Sphaerium*	4
*Unio*	6
**Oligochaeta**	
Tubificidae	1
**Hirudinea**	
*Haemopis*	4
*Glossiphonia*	4

The FBI mainly reflects the influence of the pollution tolerance level and number of different families of macroinvertebrates. The mean values of the MMIF in 2011 and 2018 were 0.57 and 0.69, respectively. The MMIF values (range of 0.35–0.75) were the highest at monitoring sites FH8-FH10 in 2011, and ranged from 0.5 to 0.85 in 2018 and were highest at FH1 and FH8-FH10. The mean FBI values in 2011 (range of 4.52–6.65) and 2018 (range of 4.23–5.99) were 5.38 and 5.21, respectively, and the values were highest at FH2 and FH5.

#### River habitat assessment in the Fan River basin

The physical habitat quality of aquatic ecosystems (flow velocity, sediment, riparian zone, vegetation, land use, etc.) can be described using integrated approaches that incorporate different habitat components^6,40^. According to the habitat investigation, the entropy and entropy weight of each habitat indicator were defined and calculated by the entropy method. The entropy weights of the quantity of sediment in the riverbed, vegetation structural integrity and riparian land use pattern were higher than those of other indicators in 2011 (Table 2). The entropy weights of the riparian width, vegetation coverage, vegetation structural integrity and riparian land use pattern were highest in 2018 (Table 2).

**Table 2:** The entropies and weight values of river habitat assessment indicators.

Assessing indicators	2011		2018	
	Entropy	Weight	Entropy	Weight
Flow velocity and state	0.93111	0.06938	0.93979	0.08959
Water quantity	0.93111	0.06938	0.93111	0.10252
Quantity of sediment in riverbed	0.77959	0.22197	0.93527	0.09633
Sediment coverage rate of silt	0.90309	0.09759	0.94072	0.08822
Riparian type	0.95424	0.04608	0.93527	0.09633
Erosion degree of riparian	0.93193	0.06855	0.93111	0.10252
Riparian width	0.92012	0.08045	0.92867	0.10615
Vegetation coverage	0.92867	0.07184	0.92869	0.10611
Vegetation structural integrity	0.84510	0.15599	0.92869	0.10611
Riparian land use pattern	0.88238	0.11845	0.92869	0.10611

The RHQI ranged from 0.36 to 0.97 in 2011 (Table 3). Based on the classification criteria of river habitat quality (Table 4), sampling stations with excellent, good, moderate and poor grades accounted for 40.0%, 10.0%, 30.0% and 20.0% of all stations, respectively. There were abundant types of bottom sediment in the riverbed at the sampling stations with excellent and good grades, and the riparian vegetation structure was relatively complete. The riparian land use patterns at these stations were mostly composed of woodlands, shrubs, grasslands and natural wetlands, with only a small amount of agricultural land. At the moderate stations, the types of bed sediment and the structural integrity of vegetation decreased, and the riparian land use pattern changed. Specifically, the proportion of woodlands decreased, and that of agricultural land increased. At the poor stations, the proportion of pebbles in the riverbed sediment decreased, and the proportions of debris and silt increased. Moreover, grasslands and agricultural land were the main vegetation types in the riparian zone.

**Table 3:** The synthetic results of river habitat assessment.

Sampling stations	2011		2018	
	River habitat quality Index	Class of river habitat quality	River habitat quality Index	Class of river habitat quality
FH1	0.82	Excellent	0.86	Excellent
FH2	0.57	Moderate	0.55	Moderate
FH3	0.50	Moderate	0.54	Moderate
FH4	0.37	Poor	0.45	Moderate
FH5	0.36	Poor	0.39	Poor
FH6	0.50	Moderate	0.56	Moderate
FH7	0.76	Excellent	0.80	Excellent
FH8	0.64	Good	0.80	Excellent
FH9	0.97	Excellent	0.95	Excellent
FH10	0.86	Excellent	0.85	Excellent

**Table 4:** Classification criteria of aquatic ecosystem health assessment.

Classification criteria	MMIF^6^	FBI^36^	RHQI^31^	IWPI^32^
Excellent	0.00–0.29	0–3.75	>0.75	20
Good	0.30–0.49	3.75–5.0	0.60–0.75	20–40
Moderate	0.5–0.69	5.0–5.75	0.45–0.6	40–60
Poor	0.7–0.89	5.75–7.25	0.3–0.45	60–80
Bad	0.9–1.00	7.25–10.0	<0.3	80–100

The RHQI in 2018 (range of 0.39–0.95, Table 3) with excellent, moderate and poor grades accounted for 50.0%, 40.0% and 10.0%, respectively. Compared with 2011, the quality of river habitat was improved. In the evaluation of river habitat quality, the indicators with larger entropy weights changed, and the weights of riparian width and vegetation coverage increased. The overall change of riparian land use pattern was not significant. Except in FH5, the riparian width and vegetation coverage increased obviously in the Fan River basin.

#### Water quality assessment in the Fan River basin

Water quality is an important component of aquatic ecological health and has an important impact on the survival of aquatic organisms^41^. The descriptive statistics for the physical-chemical parameters in 2011 and 2018 were shown in Table 5. The average values of the TP, NH_4_^+^-N, COD, BOD_5_ and DO concentrations in 2011 were 0.15 mg/L, 0.55 mg/L, 16.30 mg/L, 3.77 mg/L and 7.59 mg/L, respectively, with relatively little spatial variability. Except for DO and TP, the average values of the other water quality parameters in 2018 were lower than those in 2011, and the NH_4_^+^-N concentration exhibited more spatial variability (coefficient of variation of 86.23). The NH_4_^+^-N concentration displayed a significant difference between 2011 and 2018 according to the Mann–Whitney U test (*p* < 0.05).

**Table 5:** Characteristics of physical-chemical parameters.

Parameters	2011				2018			
	Mean	SD	CV	Range	Mean	SD	CV	Range
TP (mg/L)	0.15	0.03	22.87	0.11–0.22	0.15	0.05	34.25	0.05–0.21
NH_4_^+^-N (mg/L)	0.55	0.15	26.62	0.34–0.83	0.09	0.07	86.23	0.03–0.26
COD (mg/L)	16.30	2.63	16.12	13.00–22.00	14.60	2.71	18.53	11.50–19.00
BOD_5_ (mg/L)	3.77	0.43	11.42	3.27–4.48	3.68	0.84	22.75	2.39–4.72
DO (mg/L)	7.59	2.09	27.56	2.62–9.43	8.44	0.97	11.46	7.03–10.02

The IWPI is a synthetic water quality index that aggregates some water quality parameters through a weighted arithmetic mean function. The method obtains IWPI values through the comprehensive combination of the water pollution index and entropy weights^42^. The original matrix of water quality monitoring data was initialized and sorted, and the entropy and entropy weight of each parameter were calculated according to formula (4). The entropy weight difference of each indicator in 2011 was smaller than that in 2018. In 2011, BOD_5_, TP, NH_4_^+^-N and COD had the large entropy weights (Table 6). In 2018, the entropy weights of TP and BOD_5_ were relatively high compared to those of the other components, followed by DO and COD (Table 6).

**Table 6:** The entropies and weight values of physical-chemical parameters.

Parameters	2011		2018	
	Entropy	Weight	Entropy	Weight
TP (mg/L)	0.92643	0.20011	0.77594	0.34636
NH_4_^+^-N (mg/L)	0.92881	0.19364	0.94381	0.08686
COD (mg/L)	0.93728	0.17061	0.90110	0.15289
BOD_5_ (mg/L)	0.89642	0.28173	0.84542	0.23896
DO (mg/L)	0.94341	0.15391	0.88683	0.17494

The IWPI was calculated based on the environmental quality standards for surface water in China^43^, the corresponding WPI limiting values^44^ and the entropy weights of water parameters. The IWPI ranges from 0 to 100, and water quality can be classified as excellent (0–20), good (20–40), moderate (40–60), poor (60–80) and bad (80–100). The IWPI results are shown in Table 7. The IWPI values ranged from 38.44 to 62.91 in 2011, except at FH2 and FH9, and the average water quality grade was moderate. In 2018, the IWPI values (range of 22.36–51.82) decreased significantly (*p* < 0.05), and the sampling stations with good and moderate grades accounted for 40.0% and 60.0% of all stations, respectively. Compared with 2011, the comprehensive water quality improved in 2018.

**Table 7:** The synthetic results of improved water pollution index.

Sampling stations	2011		2018	
	Improved water pollution index	Class of water quality	Improved water pollution index	Class of water quality
FH1	47.73	Moderate	22.36	Good
FH2	62.91	Poor	42.49	Moderate
FH3	50.16	Moderate	48.10	Moderate
FH4	49.09	Moderate	48.27	Moderate
FH5	52.18	Moderate	51.82	Moderate
FH6	50.65	Moderate	26.07	Good
FH7	41.30	Moderate	40.79	Moderate
FH8	40.09	Moderate	33.02	Good
FH9	38.44	Good	34.89	Good
FH10	42.42	Moderate	41.75	Moderate

### Aquatic ecosystem health assessment in the Fan River basin

Through the establishment of a fuzzy matrix of the four evaluation indices and the calculation of the weight matrix, the membership degree of each sampling station at the five river health assessment grades, i.e., excellent, good, moderate, poor and bad, were calculated. Then, fuzzy comprehensive evaluation results were obtained through a membership degree comparison. The fuzzy comprehensive evaluation method transforms the measured values of each index into quality values reflecting the health degree through a functional relationship; this approach overcomes artificial clarity issues and has a clear calculation principle^45–48^.

Based on such a classification, 5 sampling stations (FH1, FH7-FH10) exhibited good grades (50.0% sites), 4 stations (FH3-FH6) had moderate grades (40.0%), and 1 station (FH2) had a poor grade (10.0%) in 2011 (Table 8). The sampling stations with good grades were mainly located near the Provincial Nature Reserve of the Fan River. The stations with moderate and poor grades were near villages and towns in the Fan River basin, with more farmland, livestock and poultry breeding around them. Domestic waste, agricultural pollutants^49^, and livestock and poultry breeding can affect the water quality^50^, the composition of the benthic macroinvertebrate community, and the health of aquatic ecosystems.

**Table 8:** Fuzzy comprehensive evaluation of aquatic ecological health at different sites in 2011.

Sampling stations	Excellent	Good	Moderate	Poor	Bad	Health grade
FH1	0.1719	0.4647	0.3608	0.0000	0.0000	Good
FH2	0.0000	0.1396	0.4286	0.4321	0.0000	Poor
FH3	0.0000	0.3024	0.6233	0.0743	0.0000	Moderate
FH4	0.0000	0.2469	0.5861	0.1670	0.0000	Moderate
FH5	0.0000	0.1445	0.6296	0.2259	0.0000	Moderate
FH6	0.0000	0.0891	0.8928	0.0181	0.0000	Moderate
FH7	0.1935	0.5611	0.2454	0.0000	0.0000	Good
FH8	0.1170	0.8281	0.0548	0.0000	0.0000	Good
FH9	0.3650	0.6350	0.0000	0.0000	0.0000	Good
FH10	0.2932	0.6714	0.0354	0.0000	0.0000	Good

In 2018, the river health status of the Fan River basin was improved (Table 9). The health grade in FH1 and FH9 achieved excellent. There were 5 sampling stations (FH3, FH6-FH8 and FH10) with good grade, accounted for 50.0% of the total stations. The number of moderate grade stations (FH2 and FH4-FH5) reduced. There was no poor or bad grade station.

**Table 9:** Fuzzy comprehensive evaluation of aquatic ecological health at different sites in 2018.

Sampling stations	Excellent	Good	Moderate	Poor	Bad	Health grade
FH1	0.5047	0.4953	0.0000	0.0000	0.5047	Excellent
FH2	0.0000	0.4723	0.5134	0.0143	0.0000	Moderate
FH3	0.0000	0.5016	0.4984	0.0000	0.0000	Good
FH4	0.0000	0.3108	0.6892	0.0000	0.0000	Moderate
FH5	0.0000	0.0924	0.7653	0.1423	0.0000	Moderate
FH6	0.1096	0.6158	0.2746	0.0000	0.1096	Good
FH7	0.1935	0.7117	0.0948	0.0000	0.1935	Good
FH8	0.4692	0.5145	0.0163	0.0000	0.4692	Good
FH9	0.6319	0.3681	0.0000	0.0000	0.6319	Excellent
FH10	0.3294	0.6462	0.0244	0.0000	0.3294	Good

### Interrelationship among river habitat, water quality and benthic macroinvertebrates

The characteristics of benthic macroinvertebrates, river habitat conditions, water quality parameters and the interrelationships among these factors were investigated to identify the main factors that influence river ecological health. The correlation analysis in 2011 (Table 10) showed that the RHQI value was closely associated with the MMIF, FBI and IWPI (r values of 0.629, −0.815 and −0.675, respectively). Good FBI and IWPI values are small, and good MMIF and RHQI are large. Both the FBI and IWPI were negatively correlated with the RHQI, and the larger the RHQI was, the smaller the FBI and IWPI values were. The larger River Habitat Quality Index (RHQI) means a better habitat quality. Under this condition, the sensitive species and proportion of benthic macroinvertebrates increased, and the Family Biotic Index (FBI) was small (a higher FBI value indicates a larger percentage of tolerance species, while a lower FBI value indicates a larger percentage of sensitive species). Under favorable habitat conditions, the interception and filtration of the riparian zone resulted in less pollutants entering the river, and the Improved Water Pollution Index (IWPI) value was lower (the smaller the IWPI value, the better the water quality). The MMIF was positively correlated with the RHQI, and the better the habitat quality, the greater the MMIF of the benthic macroinvertebrates. Flow velocity and state and riparian land use pattern of river habitat quality were closely related to the MMIF (r = 0.798, r = 0.754), FBI (r = −0.838, r = −0.822) and IWPI (r = −0.811, r = −0.737). The more changes in flow velocity and state and the higher the score of the riparian land use pattern, the higher the MMIF and the lower the FBI and IWPI values. The quantity of sediment in the riverbed was positively correlated with the MMIF (r = 0.667); therefore, the richer the abundance of sediment species, the higher the MMIF value. The riparian width was negatively correlated with the FBI (r = −0.840, *p* < 0.01) and IWPI (r = −0.659, *p* < 0.05). Increased riparian width was conducive to the survival of low-tolerant macroinvertebrates and the improvement of water quality. The IWPI was significantly correlated with the MMIF and FBI (r = −0.844, r = 0.915). Water quality is an important factor that affects the characteristics of benthic macroinvertebrates^23^. When the water environment is in good condition, sensitive benthic macroinvertebrates can survive, and if the water is seriously polluted, the number of tolerant species will often increase^51,52^. Of the water quality parameters, TP was the main factor that influenced the MMIF and FBI. The correlation coefficients between TP and the MMIF and FBI were −0.789 and 0.830 (*p* < 0.01), respectively.

**Table 10:** Spearman correlation between metrics selected for aquatic ecosystem health in 2011.

	MMIF	FBI	RHQI	IWFI
**RHQI**	0.629^*^	−0.815^**^	1.00	−0.675^*^
Flow velocity and state	0.798^**^	−0.838^**^	0.823^**^	−0.811^**^
Water quantity	−0.213	−0.208	0.272	−0.235
Quantity of sediment in riverbed	0.667^*^	−0.55	0.745^*^	−0.495
Sediment coverage rate of silt	−0.571	0.609	−0.218	0.606
Riparian type	−0.293	0.522	−0.407	0.522
Erosion degree of riparian	0.484	−0.494	0.594	−0.292
Riparian width	0.626	−0.840^**^	0.875^**^	−0.659^*^
Vegetation coverage	0.381	−0.243	0.358	−0.257
Vegetation structural integrity	−0.153	−0.19	0.572	0.038
Riparian land use pattern	0.754^*^	−0.822^**^	0.874^**^	−0.737^*^
**IWFI**	−0.844^**^	0.915^**^	−0.675^*^	1.00
TP	−0.789^**^	0.830^**^	−0.584	0.758^*^
NH_4_^+^-N	0.526	−0.236	0.128	−0.297
COD	−0.437	0.55	−0.486	0.718^*^
BOD_5_	−0.495	0.552	−0.395	0.782^**^
DO	0.581	−0.079	−0.067	−0.236

^*^p < 0.05, **p < 0.01.

Based on benthic macroinvertebrates, river habitat indicators and water quality parameters in 2018, the relationships among them were assessed (Table 11). RHQI was significantly correlated with MMIF, FBI and IWPI (r values of 0.920, −0.888 and −0.772, respectively, *p* < 0.01). Riparian land use pattern was still the main influencing factor for benthic macroinvertebrates (MMIF r = 0.913, FBI r = −0.905, *p* < 0.01) and water quality (r = −0.663, *p* < 0.05). Habitat indicators of riparian type and vegetation structural integrity showed significant correlations with MMIF (*p* < 0.05) and FBI (*p* < 0.01). Erosion degree of riparian and vegetation coverage exhibited significant correlations with MMIF (*p* < 0.05) and IWFI (r = −0.838, *p* < 0.01, r = −0.683, *p* < 0.05). IWFI, TP and COD were closely related to MMIF (r values of −0.820, −0.838 and −0.905, respectively, *p* < 0.01) and significantly related to FBI (r values of 0.627, 0.664 and 0.760, respectively, *p* < 0.05). There was a significant correlation between DO and MMIF (r = 0.697).

**Table 11:** Spearman correlation between metrics selected for aquatic ecosystem health in 2018.

	MMIF	FBI	RHQI	IWFI
**RHQI**	0.920^**^	−0.888^**^	1.00	−0.772^**^
Flow velocity and state	0.55	−0.389	0.547	−0.311
Water quantity	0.02	−0.509	0.171	0.221
Quantity of sediment in riverbed	0.21	−0.166	0.129	−0.097
Sediment coverage rate of silt	0.51	−0.629	0.586	−0.225
Riparian type	0.693^*^	−0.874^**^	0.751^*^	−0.499
Erosion degree of riparian	0.740^*^	−0.409	0.659^*^	−0.838^**^
Riparian width	0.304	−0.18	0.403	−0.277
Vegetation coverage	0.761^*^	−0.603	0.746^*^	−0.683^*^
Vegetation structural integrity	0.669^*^	−0.844^**^	0.877^**^	−0.543
Riparian land use pattern	0.913^**^	−0.905^**^	0.877^**^	−0.663^*^
**IWFI**	−0.820^**^	0.627^*^	−0.772^**^	1.00
TP	−0.838^**^	0.664^*^	−0.790^**^	0.988^**^
NH_4_^+^-N	−0.428	0.273	−0.432	0.648^*^
COD	−0.905^**^	0.760^*^	−0.851^**^	0.693^*^
BOD_5_	−0.624	0.248	−0.541	0.867^**^
DO	0.697^*^	−0.455	0.62	−0.612

^*^p < 0.05, **p < 0.01.

Additionally, the correlation among the biological indicators, ecological traits and physicochemical parameters was determined to evaluate their diagnostic power and provide managers with more information about the river. Through correlation analysis, it was concluded that the RHQI, IWFI and biological indices of benthic macroinvertebrates were highly correlated. Many studies have indicated that water pollution and habitat destruction can negatively affect the benthic macroinvertebrate community structure^6,53^. As the three important components of aquatic ecosystem health, the changes in each parameter will affect other parameters and have a notable impact on the state of aquatic ecosystem health^54^. The aquatic ecological investigations in 2011 and 2018 were conducted at the end of the 11th and 12th Five-Year Plans of major science and technology programs for water pollution control and treatment in China, respectively. During the subsequent program of water pollution control and treatment, measures such as strengthening village pollution control, limiting the disorderly discharge of pollutants, and centralized treatment were implemented in the Fan River basin. The improvement of water quality in 2018 was conducive to increasing the abundance and diversity of benthic macroinvertebrates, especially those that are sensitive and have low pollution tolerance levels.

In terms of river habitat, land use patterns changed. Changes in the riparian land use pattern can affect the river habitat, nutrient levels, pollutant levels, sediment load and litter load entering rivers, thus affecting the water quality and the survival of benthic macroinvertebrates^3,4,20^. In both 2011 and 2018, the riparian land use pattern was the important factor that affected benthic macroinvertebrates and water quality in the Fan River basin. The major riparian land use changed from farmland to wetlands, grasslands or a shrub-grass combination, and the riparian width increased; therefore, soil erosion was reduced. The application of pesticides and chemical fertilizers was also reduced, and the levels of nutrient pollutants entering rivers through surface runoff decreased. Moreover, pollution control by riparian vegetation improved. The improvements in water quality and river habitat were beneficial for optimizing the community structure of benthic macroinvertebrates, increasing the proportions of groups with low pollution tolerances (e.g., Ephemeroptera, Plecoptera and Tricoptera), and yielding healthy MMIF and FBI results. Therefore, the aquatic ecosystem health of the Fan River basin in 2018 was better than that in 2011. In evaluating these associations, we gathered the relevant information and found that the proposed combined assessment approach can be used to make decisions about ecological management and the conservation of river ecosystems. This study also had some limitations. The indices adopted are mainly suitable for fording rivers in mountainous areas, generally the upper reaches of rivers and the main tributaries, and not applicable for large rivers that cannot be forded and areas with poor habitat indexes in downstream areas.

## Materials and Methods

### Study area

**Figure 3:**
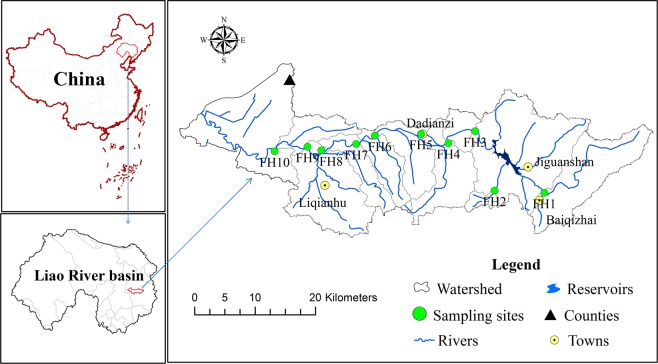
Locations of sampling stations.

The Fan River, a main tributary of the Liao River with a total length of 102 km, is located in Northeast China. The Fan River basin is located between N 42°00′00″~42°15′00″ and E 123°37′13″~124°30′25″, with a catchment area of 4785 km^2^. The study area has a temperate monsoon climate, and the average annual precipitation and temperature are 668.5 mm and 7.3 °C, respectively. It is cold and dry in the winter (December, January and February) and hot and rainy in the summer (June, July and August). The geomorphological features of the basin include low hills, plains and plateaus. There are no serious industrial pollution sources in the region, the livestock industry in the upstream region is relatively developed, and some mineral resources are distributed in the basin. The middle reaches pass through towns and farmland areas with approximately 40 km^2^ of agricultural land and some characteristic tourism. The downstream areas are mainly woodlands with a few scattered villages and farmland areas and little human disturbance. Ten sampling stations in the Fan River basin were selected for the investigation of benthic macroinvertebrates, habitat and water quality in August of 2011 and 2018 (Fig. 3). For comparison, the samples were collected in the same season and month.

### Sample collection and river habitat investigation

Benthic macroinvertebrates were sampled at the 10 sites. A net (size of 0.3 × 0.4 m, pore diameter of 425 µm) was placed in the set position, and the lower frame was fixed on the river bottom. Individual rocks were picked up and washed over a sampling barrel, and the substrates were disturbed so that macroinvertebrates flowed into the net. Then, the organisms in the net were washed into the sampling barrel, passed through a 40-mesh sieve, and preserved in 70% ethanol. The collected samples were sorted, counted and identified according to the relevant identification data^55,56^.

The investigation of habitat was conducted based on the evaluation indices and methods for river habitats^34^. There are 10 habitat indicators that reflect the quality of habitat, and a high score indicates a high-quality habitat. The physicochemical parameters of water quality, such as total phosphorous (TP), ammonia nitrogen (NH_4_^+^-N), 5-day biochemical oxygen demand (BOD_5_), potassium bichromate index (COD) and dissolved oxygen (DO) concentrations, were measured. The sampling, preservation and analytical procedures were performed according to the national standard methods of China^57^.

### Health assessment method

To assess and classify aquatic ecosystem health based on the macroinvertebrate indices, the MMIF^32^ and FBI were selected. The MMIF index of the macroinvertebrates includes Taxa Richness, Number of EPT Taxa, Number of other (i.e. non-EPT) Sensitive Taxa, Shannon–Wiener Diversity Index and the Mean Tolerance Score. The score of the corresponding index was calculated according to the river type and the scoring criteria for calculating the Multimetric Macroinvertebrate Index Flanders. These five metric scores are summed and subsequently divided by 20 to obtain the MMIF index, which ranges from 0 to 1, indicating very poor ecological quality to very good ecological quality.

1 $${\rm{Family}}\,{\rm{Index}}:{\rm{FBI}}=\sum _{i={\rm{1}}}\frac{{{\rm{n}}}_{i}{t}_{i}}{N}$$

where n_*i*_ is the number of individuals of *i* family, t_*i*_ is the tolerance of family *i*, and *N* is the total number of organisms in the sample.

In the river habitat quality assessment, the RHQI method was applied^34^. First, we normalized the score of each habitat indicator, calculated the corresponding entropy weight^35,41^, and obtained the comprehensive habitat quality score. The entropy weight of each indicator is calculated through the following process:

The entropy of the *i*th indicator is defined as

2 $${H}_{i}=-\,k\mathop{\sum }\limits_{j=1}^{n}{f}_{ij}\mathrm{ln}\,{f}_{ij},i=1,2,\cdots ,m$$

in which *m* and *n* represent *m* indicators and *n* evaluating objects, respectively, ${f}_{ij}={r}_{ij}/\mathop{\sum }\limits_{j=1}^{n}{r}_{ij}$, *k* = 1/ln *n*, and suppose that when f_*ij*_ = 0, f_*ij*_lnf_*ij*_ = 0.

The entropy weight of the *i*th indicator:

3 $${w}_{i}=\frac{1-{H}_{i}}{m-\mathop{\sum }\limits_{i=1}^{m}{H}_{i}}$$

in which ≤wi ≤1, $\mathop{\sum }\limits_{i=1}^{m}{w}_{i}=1$.

The water quality was assessed based on the IWPI method:

4 $${\rm{IWPI}}=\mathop{\sum }\limits_{i=1}^{n}\,{w}_{i}\ast [{{\rm{WPI}}}_{l}(i)+\frac{C(i)-{C}_{l}(i)}{{C}_{h}(i)-{C}_{l}(i)}\times 20]$$

where *w*_*i*_ is the weight of the *i*th indicator, which is calculated by the entropy method^35,41^; WPI_*l*_(*i*) is the corresponding minimum WPI value of the *i*th indicator^43^; *C*(*i*) is the monitoring value of the *i*th indicator; and *C*_*l*_(*i*) and *C*_*h*_(*i*) are the low and high limiting values of the *i*th indicator based on the environmental quality standards for surface water in China (GB3838-2002). These values are specific to DO, and ${C}_{h}(i)\le C(i)\le {C}_{l}(i)$.

To evaluate the status of aquatic ecosystem health, a fuzzy comprehensive evaluation method was applied. The fuzzy comprehensive evaluation method is based on determining the evaluation grade standard and weight of the “evaluation factors”, using the principle of the fuzzy set transformation, describing the fuzzy boundary of each factor by the membership degree, constructing the fuzzy evaluation matrix, and finally determining the grade of the evaluation object through a multi-layer compound operation. According to the classification criteria (Table 10) and the calculated MMIF, FBI, RHQI and IWPI values, the membership degrees of different grades were obtained. A fuzzy matrix (*R*) was formed, and the weight of each index was calculated by the method of over-standard weighting. After normalization, the weight matrix (*A*) was constructed. The comprehensive evaluation results of the aquatic ecosystem health (matrix *B*) were obtained by matrix operations (*B* = *A* · *R*).

The fuzzy matrix (R):

5 $$R=[\begin{array}{cccc}{r}_{11} & {r}_{12} & \cdots  & {r}_{1n}\\ {{\rm{r}}}_{21} & {{\rm{r}}}_{22} & \cdots  & {r}_{2n}\\ \vdots  & \vdots  & \cdots  & \vdots \\ {r}_{m1} & {r}_{m2} & \cdots  & {r}_{mn}\end{array}]$$

6 $${\rm{The}}\,{\rm{weight}}\,{\rm{matrix}}\,(A):{a}_{i}=\frac{{w}_{i}}{\sum {w}_{i}},A=\{{a}_{1},{a}_{2},\cdots ,{a}_{m}\}$$

*w*_*i*_ is calculated using the formula:

7 $${w}_{i}=\frac{{x}_{i}}{{s}_{i}}$$

or

8 $${w}_{i}=\frac{{s}_{i}}{{x}_{i}},$$

where *x*_*i*_ is the actual value of each index, and *s*_*i*_ is the average of the different levels of each index. The smaller the value, the better the index is when calculated by Eq. (7), and vice versa when calculated by Eq. (8).

matrix B:

9 $$B=A\cdot R=\{{a}_{1},{a}_{2},\cdots ,{a}_{m}\}\cdot [\begin{array}{cccc}{r}_{11} & {r}_{12} & \cdots  & {r}_{1n}\\ {{\rm{r}}}_{21} & {{\rm{r}}}_{22} & \cdots  & {r}_{2n}\\ \vdots  & \vdots  & \cdots  & \vdots \\ {r}_{m1} & {r}_{m2} & \cdots  & {r}_{mn}\end{array}]=\{{b}_{1},{b}_{2},\cdots ,{b}_{m}\}$$

According to the principle of the maximum membership degree, the grade with the maximum *b* value represents the aquatic ecosystem health grade at this point. The status of the aquatic ecosystem health was determined by a membership degree comparison. The basic data statistics were analyzed in Microsoft Excel 2010, and the multivariate statistical analyses and Mann–Whitney U test were conducted in SPSS 19.0.

### Ethics statements

No specific permits were required for the described field studies; the sampling did not cause any disturbance to the environment or to the protected species at the sampling sites.
